# BMP and retinoic acid regulate anterior–posterior patterning of the non-axial mesoderm across the dorsal–ventral axis

**DOI:** 10.1038/ncomms12197

**Published:** 2016-07-13

**Authors:** Richard W. Naylor, Lauren Brilli Skvarca, Christine Thisse, Bernard Thisse, Neil A. Hukriede, Alan J. Davidson

**Affiliations:** 1 Department of Molecular Medicine and Pathology, University of Auckland, Auckland 1142, New Zealand; 2 Department of Developmental Biology, University of Pittsburgh, Pittsburgh, Pennsylvania 15213, USA; 3 Center for Critical Care Nephrology, University of Pittsburgh, Pittsburgh, Pennsylvania 15213, USA; 4 Department of Cell Biology, University of Virginia, Charlottesville, Virginia 22903, USA

## Abstract

Despite the fundamental importance of patterning along the dorsal–ventral (DV) and anterior–posterior (AP) axes during embryogenesis, uncertainty exists in the orientation of these axes for the mesoderm. Here we examine the origin and formation of the zebrafish kidney, a ventrolateral mesoderm derivative, and show that AP patterning of the non-axial mesoderm occurs across the classic gastrula stage DV axis while DV patterning aligns along the animal–vegetal pole. We find that BMP signalling acts early to establish broad anterior and posterior territories in the non-axial mesoderm while retinoic acid (RA) functions later, but also across the classic DV axis. Our data support a model in which RA on the dorsal side of the embryo induces anterior kidney fates while posterior kidney progenitors are protected ventrally by the RA-catabolizing enzyme Cyp26a1. This work clarifies our understanding of vertebrate axis orientation and establishes a new paradigm for how the kidney and other mesodermal derivatives arise during embryogenesis.

## Abstract (web summary)

It is unclear how the dorsal-ventral (DV) and anterior-posterior (AP) axes established in the gastrula affect tissues. Here, the authors show that in zebrafish kidney development, with regard to non-axial mesoderm, the classic DV axis corresponds to the AP axis, and is regulated by BMP and retinoic acid.

## Graphical abstract

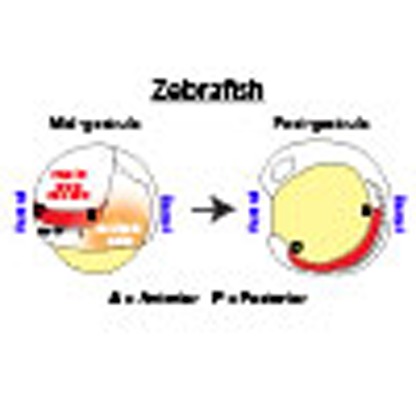

The patterning of different cell types along the dorsal–ventral (DV), anterior–posterior (AP) and animal–vegetal axes determines the organization of the embryonic body plan. In zebrafish, the mesoderm arises from an equatorial marginal zone on the yolk cell. The formation of the shield on one side breaks the radial symmetry of the marginal zone and acts as a major signalling centre for patterning. Classically, the shield marks the dorsal side of the embryo with the DV axis running equatorially to the opposite (ventral) side. Pregastrula fate mapping has shown that while cells of the shield do indeed give rise to the axial mesoderm (prechordal plate and notochord), consistent with being dorsal derivatives, the arrangement of precursors in the remaining ventrolateral mesoderm is more consistent with being aligned with the AP, and not DV, axis of the post-gastrula embryo^1^,^2^,^3^. For instance, ventrolateral mesoderm generates much of the posterior tissues including the posterior lateral plate mesoderm, pronephros, blood, posterior somites and tail. While mesoderm towards the shield region gives rise to anterior tissues such as the head mesoderm, heart and anterior trunk somites^3^,^4^,^5^. As a result, there has been uncertainty as to whether the classically held early gastrula DV axis corresponds to the actual DV axis or to the AP axis with regards to the mesoderm layout in post-gastrula embryos.

The zebrafish embryonic kidney (pronephros), comprised of two tubules that are segmented along the AP axis^6^,^7^, provides a useful tissue to help resolve the alignment of ventrolateral fates and axes. The pronephric tubules are subdivided into two anterior segments, the proximal convoluted tubule (PCT) and the proximal straight tubule (PST), and two posterior segments, the distal early (DE) and distal late (DL) segments^7^. The pronephros arises from the intermediate mesoderm, which together with the posterior lateral plate mesoderm, forms the majority of the mesodermal tissue lateral to the trunk somites (a.k.a. the posterior lateral mesoderm (PLM)). All of this mesoderm ultimately descends from the ventrolateral mesoderm of the early gastrula^3^,^5^. Given this, it is likely that a better understanding of how the pronephros is patterned along the AP axis will give insights into how the non-axial mesoderm is aligned with respect to the classic gastrula DV axis.

Bone morphogenetic proteins (BMPs) are thought to be responsible for inducing and patterning mesodermal fates along the DV axis. Traditional models propose that a morphogenic gradient of BMPs, established in part by the secretion of BMP antagonists from the shield, extends across the mesoderm with graded activity inducing different mesodermal identities^8^,^9^,^10^,^11^. For instance, low to intermediate levels have been suggested to specify kidney tissue^12^,^13^,^14^. However, despite there being a clear requirement of BMP signalling for the formation of ventrolateral mesoderm, there is little evidence that BMPs act directly as morphogens that induce tissue-specific mesodermal fates such as the kidney.

By contrast, retinoic acid (RA) acts as a morphogen that regulates the AP patterning of the zebrafish pronephros and influences the formation of other non-axial mesodermal derivatives, including the blood, heart and pectoral fin^7^,^15^,^16^,^17^,^18^. RA is synthesized from vitamin A by the activities of alcohol dehydrogenases followed by aldehyde dehydrogenases, such as Aldh1a2, and is degraded by the Cyp26 family of p450 enzymes^19^. Together, Aldh1a2 and the Cyp26 enzymes are major regulators of RA availability^20^,^21^. In zebrafish, expression of *aldh1a2* is initially found around the margin of the late blastula embryo (excluding the shield) but by late gastrulation, transcripts become highly expressed dorsally. It is around this stage that an RA gradient can first be detected emanating from the dorsal side of the embryo^21^. However, in the absence of a late gastrula fate map, the location of kidney progenitors relative to this RA source has been uncertain. Zebrafish embryos deficient in RA, either due to a mutation in *aldh1a2* or by treating gastrula-stage embryos with diethylaminobenzaldehyde (DEAB), an inhibitor of aldehyde dehydrogenases, display a ‘posteriorized' pronephros in which the distal segments are expanded at the expense of proximal fates^7^. Conversely, exogenous RA treatment dose-dependently ‘anteriorizes' the pronephros. This effect is opposite to the established role of RA during neuroectoderm patterning where RA signalling aligned with the animal–vegetal pole promotes posterior identities^22^,^23^,^24^,^25^. Together, these findings suggest a model in which kidney progenitors arising from the mesoderm during gastrulation are patterned into different anterior and posterior fates in response to RA.

In this study, we analyse the patterning of the zebrafish pronephros and perform late gastrula fate-mapping experiments to show that the classic gastrula DV axis corresponds to the AP axis with regard to the non-axial mesoderm. By examining various mesoderm markers we show that the actual DV axis of the non-axial mesoderm during gastrulation is largely aligned with the animal–vegetal pole. We demonstrate that AP patterning of the pronephros occurs in sequential phases, with BMPs first acting to establish broad anterior and posterior non-axial mesodermal territories. RA then acts downstream of BMPs, but also across the classic gastrula DV axis, such that anterior kidney fates are induced ‘dorsally' while posterior kidney progenitors are protected ‘ventrally' by Cyp26a1.

## Results

### RA induces anterior kidney fates during gastrulation

**Figure 1:**
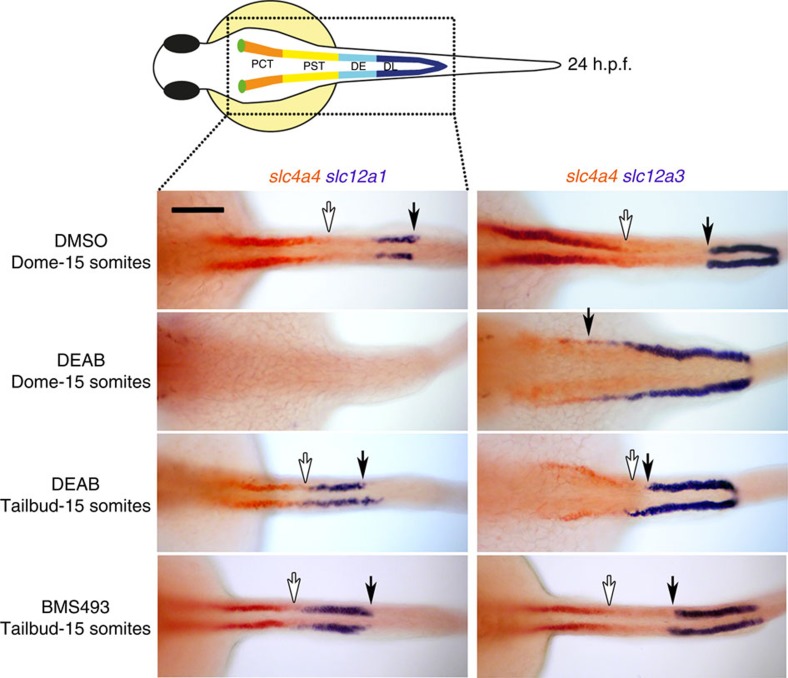
Inhibition of RA signalling affects pronephros segmentation during gastrulation. Whole-mount double *in situ* hybridization analysis at 24 hours post fertilisation (h.p.f.) for nephron segment markers *slc4a4* (red), *slc12a1* and *slc12a3* (purple) treated with BMS189453, DEAB or DMSO (vehicle control). Embryos are shown as dorsal views with anterior to the left. White arrows indicate posterior end of *slc4a4*^+^ domain and black arrows indicate the boundary between the DE and DL segments. Scale bar, 100 μm.

Prior studies showed that DEAB treatment from early gastrulation onwards results in a loss of proximal tubule formation (PCT and PST segments) and a concomitant increase in the size of the distal segments (DE and DL)^7^. We have since found that an even earlier onset of DEAB treatment, from the blastula stage (dome stage) onwards, results in a more severe phenotype with treated embryos lacking the PCT and PST segments (marked by *slc4a4* expression), as well as the DE segment (marked by *slc12a1* expression) and showing a greater expansion of the *slc12a3*^+^ DL segment (Fig. 1). By contrast, addition of DEAB or the RA receptor-α selective antagonist BMS189453 from the end of gastrulation (bud stage) onwards results in only mild AP-patterning defects (Fig. 1). On the basis of these findings we conclude that the formation of PCT, PST and DE segments are largely dependent on RA made during a pre-gastrula to late gastrula developmental window.

### Expression analysis defines different mesodermal territories

One of the challenges of studying how morphogens pattern the mesoderm has been a lack of markers that can distinguish the different mesodermal subpopulations that make up the late gastrula embryo. To help solve this, we identified the gene *zulu* (zebrafish-specific uncharacterized *LOC100537766* of unknown function), discovered from an expression screen, as a late gastrula pan-mesodermal marker^26^ (Supplementary Fig. 1). At 85% epiboly, *zulu* transcripts are expressed throughout the mesoderm, with high levels in a subset of mesoderm on the ventral side of the embryo. This expression pattern contrasts with the mesoderm markers *tbx6*, *tbx16* and *ntla*, which are expressed in nascent mesoderm but are not maintained in all derivatives^27^,^28^.

**Figure 2:**
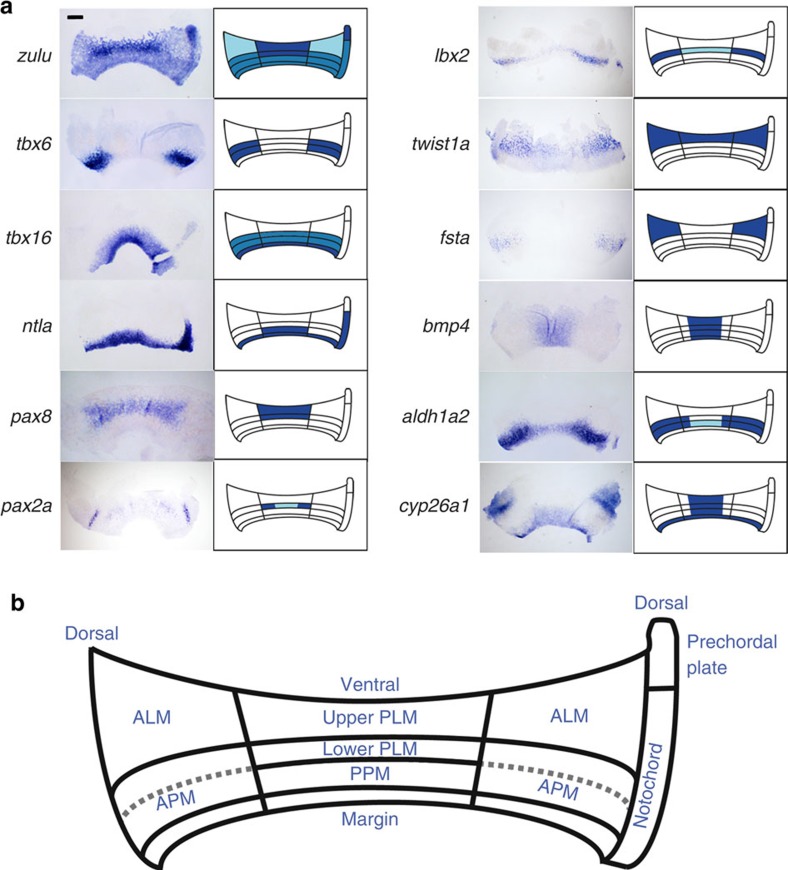
Gene expression analysis of late gastrula embryos reveals distinct ventroposterior and dorsoanterior territories. (**a**) Whole-mount *in situ* hybridization analysis for various mesodermal markers as indicated. Embryos were flat-mounted and are orientated with the animal side at the top and vegetal side at the bottom. Only mesodermal expression is highlighted with the exception of *bmp4*, which is expressed in the epiblast (expression in the hypoblast is unknown). (**b**) Schematic representation of the late gastrula mesodermal subdivisions. ALM, anterior lateral mesoderm; PPM, posterior paraxial mesoderm. Scale bar, 200 μm.

**Figure 3:**
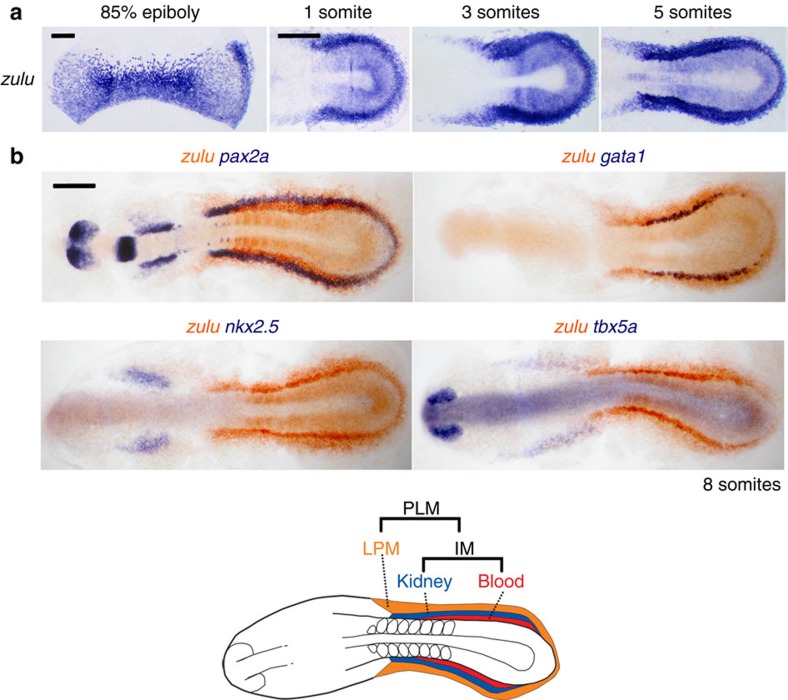
The *zulu* marker is highly expressed in the PLM. (**a**) Whole-mount stage series *in situ* hybridization detecting gene expression of *zulu* suggest the *zulu*^high^ PLM region at 85% epiboly forms the *zulu*^high^ PLM at somitogenesis stages. All embryos are flat-mounted, 85% epiboly embryos are orientated with animal pole on top and vegetal pole at the bottom, later stages are dorsal views with anterior to the left. (**b**) Whole-mount double *in situ* hybridization at the 10-somites stage to detect transcripts for *zulu* and *pax2a*/*gata1*/*nkx2.5*/*tbx5a*. All embryos are flat-mounted and shown in dorsal views with anterior to the left. Scale bars, 200 μm.

To better visualize the mesodermal domains demarcated by *zulu* expression at 85% epiboly, and to facilitate their comparison with other markers, we flatmounted stained embryos by cutting adjacent to the dorsal midline, removing the yolk and mounting under a coverslip (Fig. 2a). By similarly analysing the expression patterns of the markers *tbx6*, *tbx16*, *ntla*, *pax2a*, *pax8*, *lbx2*, *twist1a* and *fsta* we found that the mesoderm can be subdivided into different domains at 85% epiboly (Fig. 2b). Dorsally, the non-axial mesoderm can be divided into an upper domain (herein referred to as the anterior lateral mesoderm) and a lower *tbx6*^+^*/tbx16*^+^ domain of anterior trunk paraxial mesoderm (APM). On the ventral side, the mesoderm can also be separated into two domains with the lower domain expressing *ntla* and *tbx16* and likely including the precursors of the posterior trunk paraxial mesoderm^29^. The upper domain was found to express *pax2a*, *pax8* and high levels of *zulu* transcripts. Given that *pax2a*/*pax8* are early markers of the kidney^30^,^31^, we speculated that the *zulu*^high^ domain is the source of these precursors. In support of this, an expression time series is consistent with *zulu*^high^ cells giving rise to the PLM, comprising the posterior lateral plate mesoderm and the intermediate mesoderm (Fig. 3a). Double whole-mount *in situ* hybridization showed that *zulu*^high^ cells at the 10-somites stage include the intermediate mesoderm lineages: *pax2a*^+^ pronephros and *gata1*^+^ blood cells and the adjacent posterior lateral plate mesoderm, but not the *tbx5a*^+^ pectoral fin or *nkx2.5*^+^ heart fields (Fig. 3b). The expression patterns of *twist1a* and *lbx2* suggest that the PLM is further divided into upper (animal) and lower (vegetal) domains, with *twist1a* marking the upper PLM and *pax2a* and *lbx2* transcripts being restricted to the lower PLM (Fig. 2b). We found that *twist1a* and *lbx2* transcripts in the posterior of the embryo label non-overlapping domains at early somitogenesis stages, most likely corresponding to the posterior lateral plate mesoderm (*twist1a*^+^) and the intermediate mesoderm (*lbx2*^+^) (Supplementary Fig. 2). On the basis of these results we suggest that the subdivision of the PLM into posterior lateral plate and intermediate mesoderm has already occurred by 85% epiboly and takes place across the animal–vegetal pole. We also found that expression of *lbx2* was restricted to the upper APM, possibly indicating additional complexity in the patterning of the anterior somitic mesoderm (Fig. 2b).

We next examined how the expression domains of *aldh1a2* and *cyp26a1* compare with the mesodermal domains we identified at 85% epiboly. In agreement with other reports, *aldh1a2* transcripts are found throughout the margin in early gastrula embryos but became downregulated ventrally during gastrulation^32^,^33^ (Supplementary Fig. 3). By 85% epiboly, *aldh1a2* is highly expressed dorsally in the APM with lower levels extending ventrally (Fig. 2a). Transcripts for *cyp26a1* were found ventrally in the PLM and lower paraxial mesoderm domains, as well as the margin and anterior neurectoderm as previously described^25^,^34^. Within the PLM, the *cyp26a1* expression domain does not extend as laterally as the other markers expressed in this mesodermal subdivision (*zulu*, *pax2a* and *pax8*) but instead shows a more ventrally restricted pattern (Fig. 2a). Double *in situ* hybridizations revealed that expression of *cyp26a1* in the PLM included the lower *lbx2*^+^ domain (presumptive intermediate mesoderm; Supplementary Fig. 4A) while sagittal sections confirmed that *cyp26a1* transcripts on the ventral side are in the hypoblast layer (Supplementary Fig. 4B).

### Lineage labelling of RA-sensitive fates

**Figure 4:**
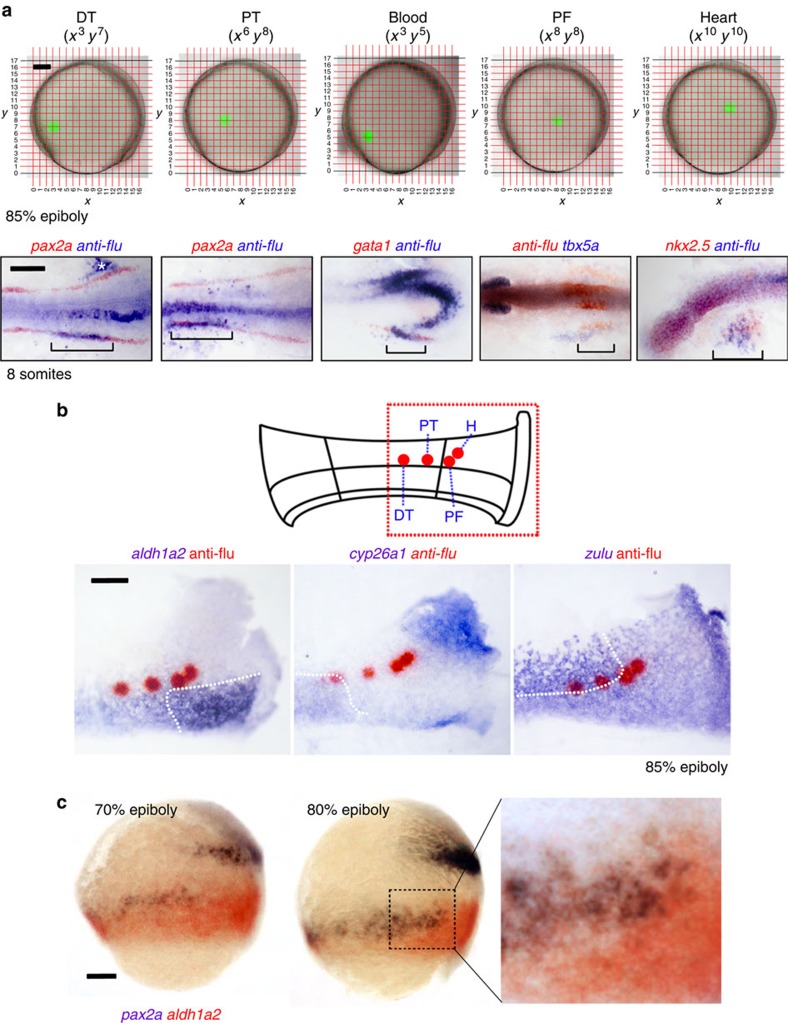
Fate-mapping analysis of late gastrula embryos show proximal tubule and pectoral fin progenitors are juxtaposed to the *aldh1a2*^high^ domain. (**a**) Cells on the left-hand side of embryos at the 85% epiboly stage were lineage-labelled at various co-ordinates on the Cartesian grid shown (embryos are orientated with animal pole towards the top and dorsal side to the right). At the 10-somites stage, co-localization of uncaged cells and kidney (*pax2a*^+^), blood (*gata1*^+^), pectoral fin (*tbx5a*^+^) and heart (*nkx2.5*^+^) tissues was determined by whole-mount double *in situ* hybridization. Labelled cells on the right-hand side of the embryo (white asterisk) are the result of the laser passing through the embryo and uncaging cells on the contralateral side. Embryos are flat-mounted with anterior to the left (note: *tbx5a* is stained purple due to being a weaker probe). (**b**) Top schematic shows a flat-mounted embryo with the highlighted region corresponding to the panels below and the positions that label progenitors of the proximal (PT) and distal (DT) tubules, pectoral fin (PF) and heart (H). Bottom panels show whole-mount double *in situ* hybridization of these uncaged populations (red) relative to the expression domains of *aldh1a2*, *cyp26a1* and *zulu* (purple) at 85% epiboly. (**c**) Lateral views of 70% (left) and 80% (centre) epiboly-stage embryos double stained for *aldh1a2* (purple/black) and *pax2a* (red) transcripts. Higher magnified view of the indicated region is shown in the right-hand panel. A, animal pole; V, vegetal pole. Schematic at the bottom represents an outline of PLM. IM, intermediate mesoderm; LPM, lateral plate mesoderm. Scale bar in **a**, 100 μm in top panels and 200 μm in lower panels. Scale bars in **b** and **c**, 100 μm.

To more precisely determine the location of the kidney progenitors within the PLM, we performed lineage labelling by photo-activation of caged fluorescein dextran in 85% epiboly-stage embryos. To ensure the targeting was reproducible, we projected a 306-point Cartesian grid onto each embryo (each point being 40 μm apart). We found that embryo size at this stage is largely constant (mean animal–vegetal pole distance was 664 μm, s.d.=5.9 μm; *n*=20). As the PLM is not morphologically discernible, we used the expression pattern of *zulu* to guide our targeting. We determined that the border between the upper *zulu*^high^ PLM and the lower paraxial mesoderm domains is coincident with a horizontal axis running midway between the animal pole (defined by the anterior extent of the prechordal plate) and the margin, which corresponds closely to the *y*^7^ and *y*^8^ axes on our fate-mapping grid. Groups of 10–20 cells were uncaged along the *y*^7^ and *y*^8^ axes at regulator coordinates across the dorsal to ventral sides of the embryo (Fig. 4a). A cluster of cells located at the (*x*^6^, *y*^8^) co-ordinate were found to give rise to anterior kidney progenitors (proximal tubule), based on co-labelling of the tracer with *pax2a* transcripts (*n*=9; Fig. 4a), while (*x*^3^, *y*^7^) labelled more posterior kidney progenitors (distal tubule; *n*=7; Fig. 4a, for transverse sections of these embryos see Supplementary Fig. 5). We also determined that *gata1*^+^ blood progenitors, *tbx5a*^+^ pectoral fin progenitors and *nkx2.5*^+^ heart progenitors, could be labelled at co-ordinates (*x*^3^, *y*^5^), (*x*^8^, *y*^8^) and (*x*^10^, *y*^10^), respectively (Fig. 4a). In addition, we confirmed that cells in the posterior trunk paraxial mesoderm domain (*x*^5^, *y*^5^), APM (*x*^10^, *y*^5^) and anterior lateral mesoderm (x^11^, y^12^) contributed to the posterior paraxial mesoderm, anterior somites and head mesoderm, respectively (Supplementary Fig. 6).

To relate the positions of the kidney progenitors to the mesodermal domains, we double-stained the embryos immediately after photo-activation for uncaged dextran and transcripts for *aldh1a2*, *cyp26a1* and *zulu* (Fig. 4b). Both anterior and posterior kidney progenitors were localized within the *zulu*^high^ domain. Anterior kidney progenitors localize closest to the *aldh1a2*^high^ dorsal domain, near the dorsalmost limit of the PLM, while the posterior kidney progenitors are located more ventrally within the subregion of the PLM that expresses *cyp26a1* (Fig. 4b). Both anterior and posterior progenitors express low levels of *aldh1a2*, and consistent with this, double *in situ* hybridizations showed that *pax2a* transcripts initiate within the weaker, more lateral, expression domain of *aldh1a2* (Fig. 4c). This orientation with anterior kidney progenitors located dorsally and posterior kidney progenitors located ventrally is consistent with the AP axis of the PLM running laterally from the ventral to dorsal sides of the embryo. These data support a model in which the concentration of RA is highest in the dorsalmost PLM, where it induces anterior kidney fates, and lower in the ventral PLM where posterior kidney fates arise.

A similar analysis performed for the pectoral fin progenitors showed that these cells are positioned more dorsally, outside of the PLM, but still adjacent to the *aldh1a2*^high^ domain. Heart progenitors were found to lie close to the pectoral fin progenitors but further towards the animal pole and therefore relatively distant to the RA source. Blood progenitors were not examined, however a subset were mapped to position (*x*^3^, *y*^5^), slightly more vegetal to posterior kidney progenitors (*x*^3^, *y*^7^), placing them in the *cyp26a1*-expressing ventral subregion of the PLM. Overall the fate-mapping data show that progenitors requiring RA for their establishment (anterior kidney and pectoral fin) are localized around the perimeter of the *aldh1a2*^high^ domain while progenitors inhibited by RA (posterior kidney, blood and heart) are found in more distal positions.

### Cyp26a1 restricts the effects of RA

**Figure 5:**
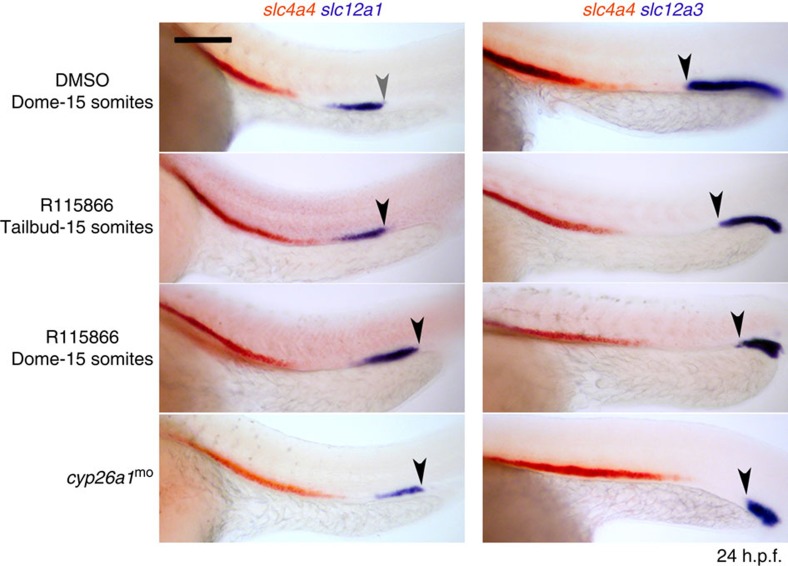
Knock down of Cyp26a1 anteriorizes the kidney. Whole-mount double *in situ* hybridization analysis at 24 hours post fertilisation (h.p.f.) for nephron segment markers *slc4a4* (orange), *slc12a1* and *slc12a3* (purple) in embryos injected with *cyp26a1* morpholino or treated with R115866 or DMSO (vehicle control). Embryos are shown as lateral views with anterior to the left. Arrowhead indicates junction between the DE and DL segments. Scale bar, 100 μm.

In certain contexts, Cyp26 enzymes are proposed to shape the RA gradient by acting as a degradative ‘sink'^24^,^35^,^36^. In doing so, Cyp26 enzymes may create a boundary that limits RA diffusion. To explore the role of *cyp26a1* in the context of kidney patterning, embryos deficient in Cyp26a1 activity were generated by morpholino-mediated knockdown and by treatment with R115866, a pan-Cyp26 inhibitor, from the late blastula stage onwards. Both methods resulted in similar ‘anteriorized' kidney phenotypes, characterized by an expansion in the anterior segments (*slc4a4*^+^ PCT and PST segments), a posteriorly displaced *slc12a1*^+^ DE segment and a concomitant reduction in the *slc12a3*^+^ DL segment (arrows, Fig. 5). Treatment of embryos with R115866 from the end of gastrulation (tailbud stage) onwards had only mild effects on renal patterning, indicating that kidney-associated Cyp26 activity is mostly required during gastrulation (Fig. 5). We conclude from these observations that Cyp26a1 is needed in the PLM during gastrulation to restrict the anteriorizing effects of RA.

### BMP regulates the *aldh1a2* and *cyp26a1* expression domains

**Figure 6:**
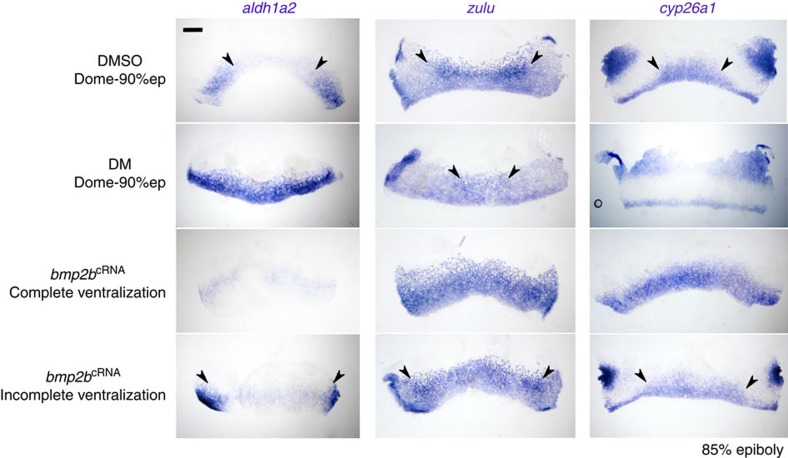
BMP signalling regulates the size of aldh1a2 and cyp26a1 and zulu expression domains. Whole-mount *in situ* hybridization analysis for *aldh1a2*, *cyp26a1* and *zulu* at the 85% epiboly stage in embryos treated with DM dihydrochloride, DMSO (vehicle control) or injected with *bmp2b* mRNA. Embryos are shown flat-mounted and orientated with the animal pole at the top and the vegetal pole at the bottom. Scale bar, 200 μm.

We next examined the effects of modulating BMP signalling, given its critical role in the formation of ventrolateral fates. To do this, embryos were treated from the late blastula stage onwards with the BMP receptor inhibitor dorsomorphin (DM) to induce a moderately ‘dorsalized' phenotype (Fig. 6). To assess PLM formation in these embryos we examined *zulu* expression at 85% epiboly and found the *zulu*^high^ PLM domain to be smaller and more ventrally restricted (Fig. 6). These results indicate that BMP signalling is needed to establish the extent of the PLM across the ventral to dorsal sides of the embryo.

**Figure 7:**
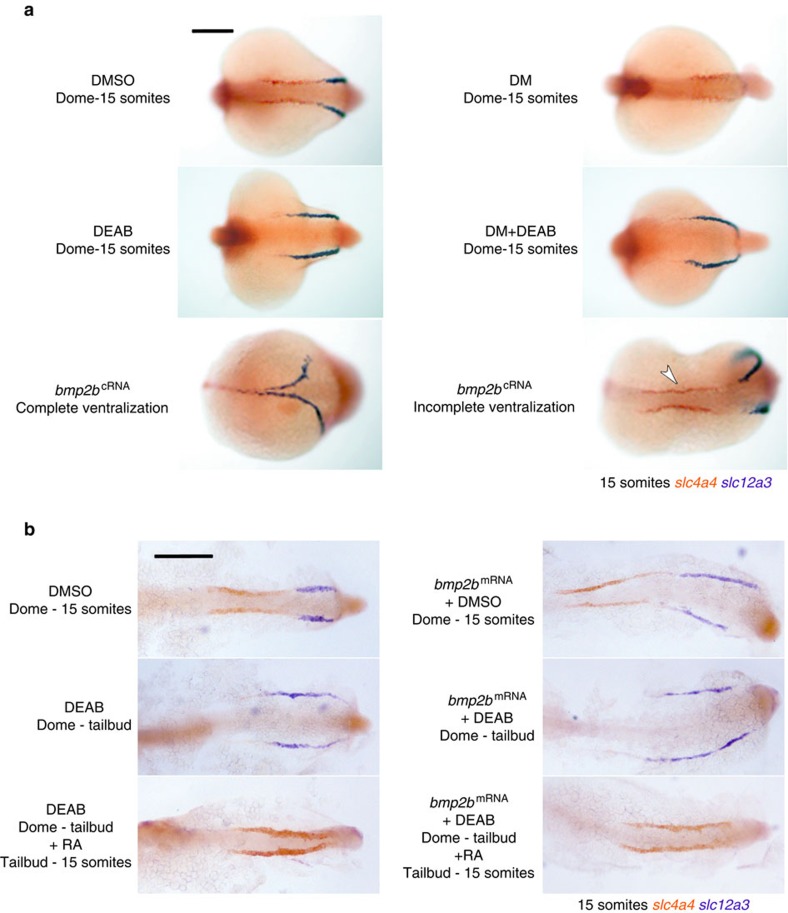
RA acts downstream of the BMP signalling pathway during kidney patterning. (**a**) Whole-mount double *in situ* hybridization analysis for nephron segment markers *slc4a4* (red) and *slc12a3* (purple) in embryos at the 15-somites stage injected with *bmp2b* mRNA or treated with DM dihydrochloride, DEAB and DMSO (vehicle control) at the indicated stages. Embryos are shown as dorsal views with anterior to the left. (**b**) Embryos were treated as shown and stopped at the 15-somites stage for double *in situ* hybridization staining for the anterior pronephros marker *slc4a4* and the distal pronephros marker *slc12a3*. All panels are dorsal views of flatmounted embryos. Scale bar, 200 μm.

To assess how modulating the BMP pathway influences RA signalling, we investigated the expression of *aldh1a2* and *cyp26a1* in DM-treated embryos. The *aldh1a2*^high^ dorsal domain was found to be expanded towards the ventral midline in DM-treated late gastrula embryos, whereas the ventral expression of *cyp26a1* is absent in these embryos (Fig. 6). These observations suggest that BMP inhibition leads to increased RA signalling in more ventral regions of the embryo by increasing *aldh1a2* expression and reducing *cyp26a1* expression. In agreement with this, DM-treated embryos display an ‘anteriorized' kidney phenotype with a severely reduced DL segment (Fig. 7a). To confirm that this phenotype was mediated downstream of BMP signalling via an increase in RA synthesis, embryos were double-treated with DM and DEAB resulting in a phenotype switch to a ‘posteriorized' (*slc12a3*^+^-only) kidney (Fig. 7a).

To examine the effects of increased BMP signalling, embryos were injected with *bmp2b* mRNA, resulting in a mix of moderately to severely ventralized embryos. In moderately ventralized embryos, the *zulu*^high^ domain was increased laterally towards the dorsal midline, whereas in severely ventralized embryos, this domain extends around the entire circumference of embryo, indicative of radial ventralization (Fig. 6). In mildly ventralized embryos, the *aldh1a2*^high^ dorsal domain is reduced, whereas it is absent in severely ventralized embryos. Conversely, the ventral expression domain of *cyp26a1* is expanded laterally in ventralized embryos (Fig. 6). These results, the reciprocal of those obtained from BMP inhibition, are consistent with increased BMP signalling leading to an enlarged *cyp26a1*^+^ PLM territory and a reduced or absent *aldh1a2*^high^ dorsal domain. Concomitant with these changes, embryos with a moderately ventralized phenotype develop abnormally long, but relatively well-patterned, kidneys, whereas severely ventralized embryos display a completely ‘posteriorized' (*slc12a3*^+^-only) kidney (Fig. 7a). Taken together, these data support a model in which BMP signalling influences kidney patterning by regulating the size of the mesodermal domains that express *aldh1a2* and *cyp26a1*.

One corollary of this model is that the effects of RA on the kidney must be occurring downstream of the mesoderm-patterning effects of BMP during gastrulation. To test this, embryos were first ventralized by *bmp2b* injection, as well as treated with DEAB from the dome stage to the end of gastrulation (bud stage) to generate ‘BMP-high/RA-low' embryos. As expected, these embryos develop ‘posteriorized' kidneys (Fig. 7b). When exogenous RA was added to ‘BMP-high/RA-low' embryos at the end of gastrulation, the kidney phenotype switched to being ‘anteriorized' (*slc4a4*^+^-only; Fig. 7b). Similarly, in embryos without altered BMP levels, the posteriorizing effect of DEAB treatment from dome to bud stage can be reversed by the addition of RA from late gastrulation (Fig. 7b). These results indicate that RA can act as late as the end of gastrulation to influence kidney fates and supports the notion that RA functions sequentially to BMP during non-axial mesoderm patterning.

### Effects of BMP and RA on cell movements

**Figure 8:**
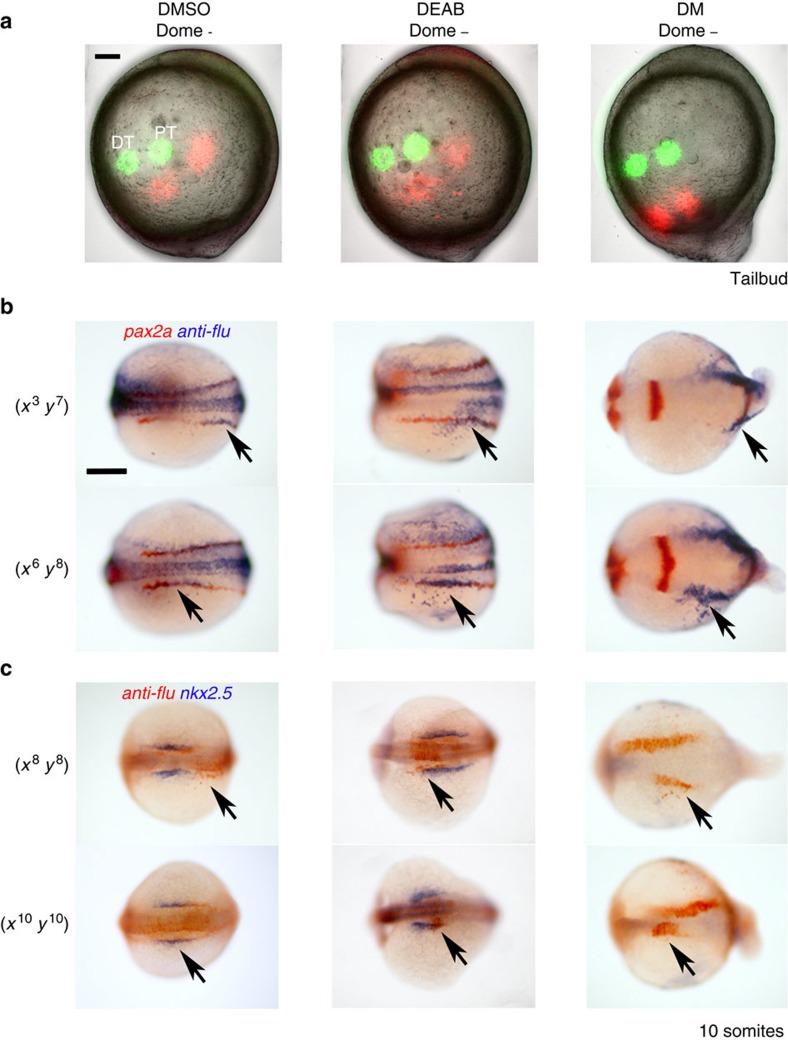
Effects of BMP and RA on cell fate and cell movement. (**a**) Embryos were lineage labelled for distal (DT) and proximal (PT) cells (green) at 85% epiboly. One hour later the positions of these cells were analysed (pseudo-coloured red) and overlayed with the initial lineage labelling. Embryos are lateral views with anterior towards the top. (**b**) Uncaging cells at (*x*^3^, *y*^7^) or (*x*^6^, *y*^8^) at 85% epiboly labelled DT and PT regions, respectively, of the *pax2a*^+^ intermediate mesoderm in DMSO- and DEAB-treated 10-somites stage embryos. In DM-treated embryos, (*x*^3^, *y*^7^) uncaging labelled anterior intermediate mesoderm and a region we predict corresponds to anterior paraxial mesoderm. DM-treated embryos uncaged at (*x*^6^, *y*^8^) labelled a region of the embryo that likely was composed solely of anterior paraxial mesoderm. Embryos are shown as dorsal views with anterior to the left. (**c**) Uncaging at (*x*^8^, *y*^8^) labelled cells just posterior to heart progenitors in DMSO control embryos at the 10-somites stage, but labelled within the posterior domain of expanded heart progenitors (*nkx2.5*^+^ cells) in DEAB-treated embryos. Similarly, (*x*^10^, *y*^10^) labelled heart progenitors in 10-somites stage DMSO control embryos, but mainly labelled the anterior region of the expanded *nkx2.5*^+^ domain in DEAB-treated embryos. In DM-treated embryos, heart progenitors were found to sparsely populate the ventral side of the embryo, and (*x*^8^, *y*^8^) and (*x*^10^, *y*^10^) labelling at 85% epiboly gave rise to progressively more anterior structures that were positioned close to the dorsal midline. Embryos are shown as dorsal views slightly oblique such that the anterior head region is in view. Scale bars, 100 μm. Arrows indicate uncaged tracer.

In addition to altering cell fates, the BMP gradient is proposed to link the DV and AP axes by regulating dorsal convergence and extension movements^37^. These movements involve the migration of ventrolateral cells towards the dorsal side of the embryo together with their intercalation, which drives elongation of the body axis^38^,^39^. The highest level of BMP signalling (encompassing 20°–30° of the ventral margin at the shield stage^37^) creates a ‘no convergence no extension zone' (NCEZ) resulting in the ventralmost cells moving vegetally, but not dorsally, and contributing to the tailbud and other posterior fates. To determine how the positions of anterior and posterior kidney progenitors relate to the NCEZ, we uncaged tracer in a 20° wedge of the ventral margin and then subsequently labelled the kidney progenitors at the 85% epiboly stage. By imaging live embryos, we found that the kidney progenitors are located well outside of the NCEZ (Supplementary Fig. 7). In addition, over a 1-h time period, we observed that the anterior (*x*^6^, *y*^8^) kidney progenitors migrate dorsally while the posterior (*x*^3^, *y*^7^) kidney progenitors move both dorsally and vegetally (*n*=3, Fig. 8a). Inhibiting BMP signalling with DM causes both populations of cells to move predominately vegetally, most likely due to the loss of directional cues conferred by the BMP gradient (Fig. 8a)^37^,^40^,^41^. Together, these findings are consistent with the notion that a BMP gradient across the ventral to dorsal sides of the embryo directs dorsal migration and that the kidney progenitors, being outside of the NCEZ, undergo differential convergence movements corresponding to their position along this gradient.

We next determined how blocking BMP signalling affects the fates of cells at 85% epiboly that would normally give rise to the anterior (*x*^6^, *y*^8^) and posterior (*x*^3^, *y*^7^) kidney by repeating our fate-mapping experiments. We found that DM treatment causes cells at position (*x*^6^, *y*^8^) to contribute to mid-trunk tissues, rather than the kidney, while cells at position (*x*^3^, *y*^7^) give rise to the anteriormost part of the *pax2a*^+^ intermediate mesoderm (Fig. 8b, see Supplementary Fig. 8a for lateral and oblique views). These results are in line with our observation that the PLM territory is ventrally restricted when BMP signalling is inhibited. As a result, cells that would normally form the posterior kidney end up being the dorsalmost cells of the PLM in DM-treated embryos and therefore populate the anterior intermediate mesoderm. Furthermore, the inhibitory effects of DM on dorsal convergence and extension provide an explanation for why the intermediate mesoderm stripes remain so posteriorly restricted in DM-treated embryos.

Significant effects of DM on the fates and positions of cells that normally contribute to pectoral fin (*x*^8^, *y*^8^) and heart (*x*^10^, *y*^10^) were also found, with both positions giving rise to upper trunk tissues, most likely anterior paraxial mesoderm, while heart progenitors formed aberrantly in lateral and ventral regions of the embryo (Fig. 8b and Supplementary Fig. 8B).

To assess whether RA also dually regulates cell movement and patterning like BMP signalling, we examined live cell movements and repeated the fate-mapping experiments in the presence of DEAB. Live imaging of uncaged cells in DEAB-treated embryos showed that the kidney progenitors undergo similar dorsal cell convergence movements as control embryos (Fig. 8a). Fate mapping of cells at positions (*x*^6^, *y*^8^) and (*x*^3^, *y*^7^) in RA-deficient embryos showed that they end up in relatively normal anterior and posterior domains of the intermediate mesoderm, respectively (Fig. 8b). Given that the kidney is ‘posteriorized' (*slc12a3*^+^-only) in these embryos (Fig. 7a), we conclude that, in the absence of RA, anterior kidney progenitors migrate to their normal position within the intermediate mesoderm but adopt a posterior fate. In addition, we found that cells at positions (*x*^8^, *y*^8^) and (*x*^10^, *y*^10^), which normally label pectoral fin and heart progenitors, respectively, both give rise to heart progenitors in DEAB-treated embryos, consistent with previous reports^15^,^16^ (Fig. 8c). Taken together, these findings further support our model that RA signalling influences the fine patterning of the non-axial mesoderm, downstream of BMPs, without having major effects on dorsal convergence movements.

## Discussion

With the advent of better fate maps in *Xenopus* it was proposed that the classic gastrula-stage DV axis may represent the AP axis, with more dorsally located cells contributing to anterior tissues^42^. While the zebrafish fate map for mesoderm has not been revisited at the same level of resolution, the fate map for the ectoderm is generally consistent with the traditional view that the AP axis is coincident with the animal–vegetal axis and not the classic gastrula stage DV axis^43^,^44^,^45^. As a result, there has been some confusion and uncertainty in the field regarding the spatial arrangement of the mesoderm along the zebrafish DV axis. In this study we make use of the kidney, with its defined segmentation pattern along the AP axis, to demonstrate that, with regard to the non-axial mesoderm, the classic DV axis of the gastrula corresponds to the AP axis while the DV axis aligns with the animal–vegetal pole. These results agree with the latest *Xenopus* fate map and with grafting experiments in zebrafish, which demonstrated that ventral grafts formed posterior mesodermal tissues while more dorsal grafts formed anterior mesodermal structures^1^. As this arrangement is the opposite to that found for the ectoderm it indicates that the AP and DV axes of the non-axial mesoderm and ectoderm are initially spatially distinct. As a result, it is impossible to consider the early embryo in terms of single AP and DV axes, which are aligned for all germ layers. Instead, the germ layer axes only become entrained following gastrulation movements.

**Figure 9:**
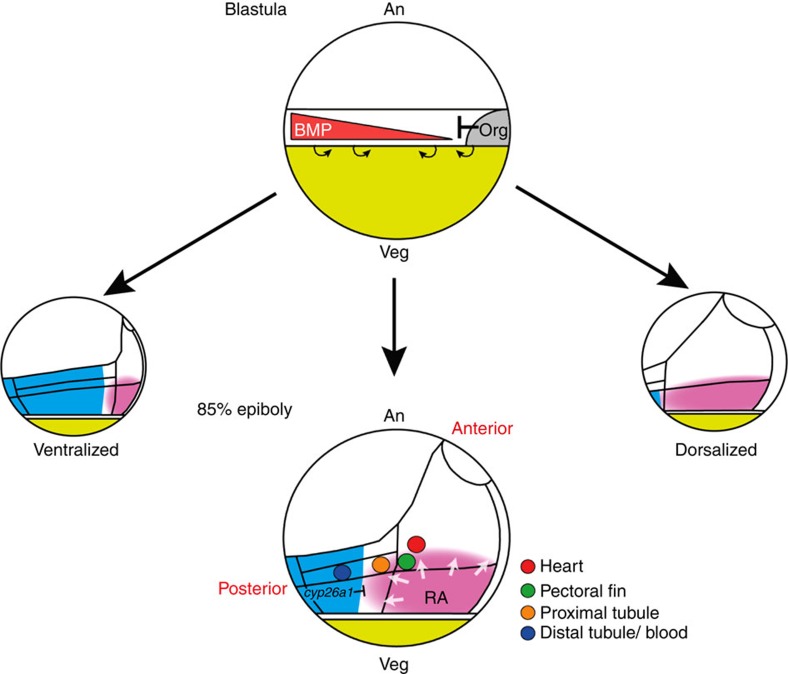
Model showing how a radial field of RA is proposed to regulate AP patterning of the mesoderm downstream of BMP signalling. Top schematic depicts a pregastrula embryo showing the equatorial mesendoderm, the organizer/shield region, and the proposed BMP activity gradient. Small curved arrows indicate the involution movements of the mesoderm at the margin. Bottom schematic depicts a late gastrula-stage embryo showing axial (prechordal plate and notochord), anterior and posterior mesodermal domains (see text for details), the positions of fate-mapped progenitors and the proposed radial field of RA relative to *cyp26a1* expression. An, animal; Veg, vegetal.

Our data indicate that BMP signalling plays a role in the initial subdivision of the non-axial mesoderm into broad territories along the AP axis. Our marker analysis identified an anterior territory (head and upper trunk mesoderm) and a posterior territory (lower trunk mesoderm and tail). Although not examined in our study, we expect the posterior territory to be further subdivided into lower trunk mesoderm and tail subdomains, as there is evidence that tail precursors are set aside during early gastrulation but patterned later (Fig. 9)^46^,^47^,^48^. We found that the respective sizes of the non-axial mesoderm territories is set by the level of BMP signalling, consistent with a large body of evidence showing that loss of BMP signalling causes a loss of posterior structures^5^,^10^,^12^,^13^,^46^,^48^,^49^,^50^,^51^,^52^,^53^,^54^,^55^,^56^,^57^,^58^,^59^. For the ectoderm, inducible BMP inhibition experiments have shown that BMP signalling acts during critical temporal intervals to pattern DV fates in a rostral to caudal progression but has little involvement in AP patterning^59^. While the mesoderm was not extensively studied, using *wt1a*, *pax2a* and *gata1* expression, as read-outs of increasingly more ‘ventral' (posterior) fates, it was revealed that BMP signalling also influences the patterning of these mesodermal tissues in a temporal fashion. As our data indicate that the AP and DV axes of the ectoderm and mesoderm are initially oriented at right angles to each other, we interpret these findings as indicating that BMP signalling in the non-axial mesoderm temporally regulates AP, but not DV, patterning. As a result, the BMP signalling gradient can be considered to function progressively over time to pattern AP identities in the non-axial mesoderm and DV tissues in the ectoderm, thus revealing common, but germ layer-specific, effects of BMPs on each axis.

The Progressive Critical Intervals model proposes that the temporal aspect of BMP signalling provides a mechanism to co-ordinate patterning such that AP and DV fates are acquired simultaneously but progressively along the embryo. While this model works well for the ectoderm, a better understanding of the temporal aspects of mesoderm patterning is needed to fully understand how this model translates to the mesoderm. Given that *twist1a* expression in posterior lateral plate mesoderm precursors initiates between 50 and 60% epiboly^60^ while transcripts for *lbx2* (ref. 61) and *pax2a* in the intermediate mesoderm appear at 70% epiboly, we speculate that DV fates may be progressively acquired along the animal–vegetal pole.

While the BMP gradient is capable of inducing specific identities, such as epidermis and neural crest, our experiments suggest that the fine AP patterning of kidney fates occurs later in gastrulation in response to RA. Notably, our observation that RA treatment at the end of gastrulation switches the kidney from being anteriorized to posteriorized in a ‘BMP-high/RA-low' embryo strongly supports the role of RA acting later in development, after the temporal effects of BMP signalling have occurred. Given that we observed little effect on dorsal convergence movements when RA synthesis was inhibited, we do not believe the patterning effects of RA on the kidney are the result of abnormal cell positioning during gastrulation. Instead, our data suggest a model in which a pool of RA is established across the PLM, with high levels on the dorsal side acting to induce anterior kidney fates while more ventrally located kidney progenitors are protected from RA by Cyp26a1. This model is in keeping with the established function of RA as an AP-patterning molecule but rather than acting along the animal–vegetal axis, as it does for hindbrain patterning^23^,^24^,^25^,^36^,^62^,^63^, it acts laterally, across the dorsal to ventral sides of the gastrula (Fig. 9). Furthermore, this role for RA does not appear specific to zebrafish, as in *Xenopus* the expression domains of *aldh1a2* and *cyp26a1* relative to the kidney anlage show a similar spatial arrangement at the end of gastrulation (Supplementary Fig. 9)^64^.

How RA signalling distinguishes the different anterior fates of the kidney and whether this occurs as a result of a morphogenic gradient remains unclear. In the hindbrain it has been proposed that Cyp26 enzymes control the shape of an RA gradient by regulating localized RA degradation^25^. The ‘read-out' of this gradient is then postulated to induce different rhombomere identities within the neuroectoderm. In our studies, knockdown of Cyp26a1 activity results in the kidney being mildly anteriorized. This observation suggests that Cyp26a1 has a role in restricting RA activity but is not likely required to form the gradient. We envision that Cyp26a1 acts in a boundary-forming capacity that protects ‘ventral' PLM progenitors from the anteriorizing effects of RA and thereby fine-tunes the proportion of anterior fates (PCT, PST and DE) induced relative to the DL fate. Because the entire kidney adopts a DL fate when RA signalling is blocked, we consider the DL segment to represent the default differentiation state of the pronephros.

In addition to kidney, the formation of erythroid cells, pectoral fin and heart is also sensitive to RA levels. With regard to red blood cells, their formation is inhibited by RA^17^, which is consistent with our fate mapping that places their progenitors in the *cyp26a1*^+^ PLM. When embryos are treated with DEAB to block RA signalling, the anterior limit of the *gata1*^+^ blood stripes shifts from being level with somite 6 to being level with somite 1 or 2 (ref. 17). This observation suggests that the more ‘dorsal' (*cyp26a1*^−^) PLM is competent to form blood but this fate is inhibited due to high RA levels. RA signalling also inhibits cardiac specification while it plays a permissive role for pectoral fin progenitors^15^,^16^,^18^. Blocking RA causes the size of the cardiac progenitor field to expand into the region where the pectoral fin develops^15^. Our fate mapping supports this interaction as both progenitor types are localized in close proximity within the anterior lateral mesoderm domain with cardiac progenitors located further away from the *aldh1a2*^high^ dorsal domain than pectoral fin progenitors. The effects of RA on the patterning of multiple tissues (kidney, blood, heart, pectoral fin and hindbrain), as well as endodermally derived tissues such as the pancreas^65^,^66^,^67^, raises the possibility that the *aldh1a2*^high^ dorsal domain acts as a ‘secondary organizer', with RA regulating cell identities in all three surrounding germ layers. When these tissues are viewed in relation to their spatial arrangement around the *aldh1a2*^high^ dorsal domain it suggests that RA should be considered to act radially, rather than simply in a planar fashion along the animal–vegetal pole as has been largely considered up until now. When viewed this way, the contradictory effects of RA as either a posteriorizing factor, such as in the hindbrain, or anteriorizing factor, as in the case of the kidney, can be explained by the position of each progenitor type relative to the presumptive radial field of RA (Fig. 9).

In summary, we have used the kidney to examine how the non-axial mesoderm is patterned during gastrulation relative to the classic DV axis. We show that the BMP and RA pathways play sequential roles as mesodermal AP-patterning molecules and are aligned across the classic gastrula stage DV axis, while actual DV patterning of the mesoderm occurs along the animal–vegetal pole. Together, these results clarify our understanding of how the mesodermal AP and DV axes arise during development and provide a new paradigm for how non-axial mesoderm derivatives, such as the kidney, form during embryogenesis.

## Methods

### Zebrafish husbandry

Zebrafish were maintained and staged according to established procedures^68^ and in accordance to the University of Auckland's Animal Ethics Committee (protocol 001343). Embryos were collected from paired matings of wild-type Tübingen (*Tg*) adults for all lineage labelling and drug treatment experiments.

### Embryo preparation for lineage labelling

A unit of 2 μg μl^−1^ of dextran DMNB-caged fluorescein biotin 10,000 molecular weight (synthesized by Invitrogen using funding from NIH R01 grant #DK078209-04S1, Tomoko Obara) was injected into embryos at the one-cell stage. Injected embryos were grown to ∼85% epiboly, dechorionated in 0.5 mg ml^−1^ pronase from *Streptomyces griseus* (Roche Diagnostics), washed three times in E3 embryo medium, then individually placed into wells on a Petri dish containing a bed of 1% agarose. Embryos were orientated to a lateral position and fitted to the 306-point grid. Uncaging was performed on the left side of all embryos by selecting a co-ordinate for a 4-s exposure to a 405-nm diode laser using a Sim Scanner on an Olympus FluoView FV1000 confocal laser scanning microscope. As the laser passes through all layers of the embryo, and out the contralateral side, both the epiblast and hypoblast are labelled on either side of the embryo. Uncaged embryos were then imaged and placed in fresh E3 embryo medium for further experimentation. Only the uncaged cells on the left-hand side of the embryo were used for the fate mapping analysis.

### Morpholinos and mRNA synthesis

A previously described morpholino oligonucleotide to *cyp26a1* (5′-CGCGAACTGATCGCCAAAACGAAA-3′)^25^,^69^ was purchased from Gene Tools LLC and resuspended in 1 × Danieau solution. One-cell embryos were injected with 1–5 nl of *cyp26a1* morpholino at 3 ng nl^−1^. Embryos were incubated at 28 °C to the desired stage, fixed in 4% paraformaldehyde (PFA) and stored in methanol. Synthetic *bmp2b* mRNA was transcribed from a *pSP64T-bmp2b* cDNA construct using the mMessage mMachine kit (Ambion). Zebrafish embryos were injected with 1–5 nl of 10 ng μl^−1^ *bmp2b* mRNA at the one-cell stage and incubated at 28 °C to the desired stage, fixed in 4% PFA and stored in methanol.

### Whole-mount *in situ* hybridization

Whole-mount *in situ* hybridization was performed as previously described^70^. Digoxigenin- or fluorescein-labelled anti-sense riboprobes were made using T7/SP6 RNA polymerase transcription kits (Roche Diagnostics). For double *in situ* hybridizations we performed two 15-min washes with 100 mM glycine, pH 2.2, to inactivate the alkaline phosphatase-mediated chromogenic reaction. The *slc4a4*, *slc12a1*, *slc12a3*, *tbx6*, *tbx16*, *ntla*, *pax8*, *lbx2*, *twist1a*, *bmp4*, *aldh1a2*, *cyp26a1*, *pax2a*, *gata1*, *nkx2.5* and *tbx5a* probes were used as previously reported^6^,^7^,^26^,^61^. The *LOC100537766*/*zulu* gene expression vector was acquired from a high-throughput expression screen^34^.

### Paraffin wax sectioning

On completion of *in situ* hybridization staining, embryos were fixed overnight in 4% PFA at 4 °C. Embryos were washed two times in PBST, then placed in plastic cryomoulds. PBST was removed and molten 1% agarose/PBS was added. Embryos were correctly orientated and left for 1 h to allow the agarose to set. The agarose block was removed from the mould and dehydrated serially in 70, 90, 95, 100% ethanol (Sigma-Aldrich) for 1 h each. The block was cleared in 50:50 ethanol:xylol (Sigma-Aldrich) then 100% xylol for 1 h each. To begin paraffin infiltration, the block was left in 50:50 xylol:HistosecParaffin (MerckMillipore) overnight at 60 °C. Hundred per cent paraffin was added and changed twice daily for 3 days. The block was then mounted on a holder and left at room temperature overnight before 6-μm sections were cut on a microtome. Sections were placed on a slide and mounted under a coverslip with Entellan (ThermoFisher).

### Embryo chemical treatments

Chemical treatments were performed at the stages stipulated in the text. DEAB (Sigma-Aldrich) was dissolved to 10 mM in dimethylsulphoxide (DMSO) and stored at −20 °C in 500-μl aliquots; embryos were treated with 1.6 μM DEAB. RA (Sigma-Aldrich) was dissolved to 10 mM in DMSO and stored at −20 °C in 50-μl aliquots; embryos were treated with 0.1 μM RA. DM dihydrochloride (SelleckChem) was dissolved to 10 mM with water and stored at −80 °C in 100-μl aliquots; embryos were dechorionated and incubated in 10 μM DM (we found chorionated embryos had greatly reduced dorsalized phenotypes when treated with DM). R115866 (Active Biochem) was dissolved to 10 mM with DMSO and stored at −20 °C in 50-μl aliquots; embryos were treated with 20 μM R115866. BMS189453 (Tocris) was dissolved to 10 mM with DMSO and stored at −20 °C in 100-μl aliquots; embryos were treated with 10 μM BMS189453. All control embryos were incubated in an equivalent DMSO concentration to the highest concentration of drug used.

### Data availability

The authors declare that the data supporting the findings in this study are available within the article or from the corresponding author on request.

## Additional information

**How to cite this article:** Naylor, R. W. *et al.* BMP and retinoic acid regulate anterior–posterior patterning of the non-axial mesoderm across the dorsal–ventral axis. *Nat. Commun.* 7:12197 doi: 10.1038/ncomms12197 (2016).

## Supplementary Material

Supplementary Information Supplementary Figures 1-9 and Supplementary References
