# Supplementary material for: BMP and retinoic acid regulate anterior–posterior patterning of the non-axial mesoderm across the dorsal–ventral axis
Source: Nat Commun. 2016 Jul 13;7:12197. doi: 10.1038/ncomms12197 (PMC4947171; doi:10.1038/ncomms12197)
Supplement: Supplementary Information — Supplementary Figures 1-9 and Supplementary References [file ncomms12197-s1.pdf]

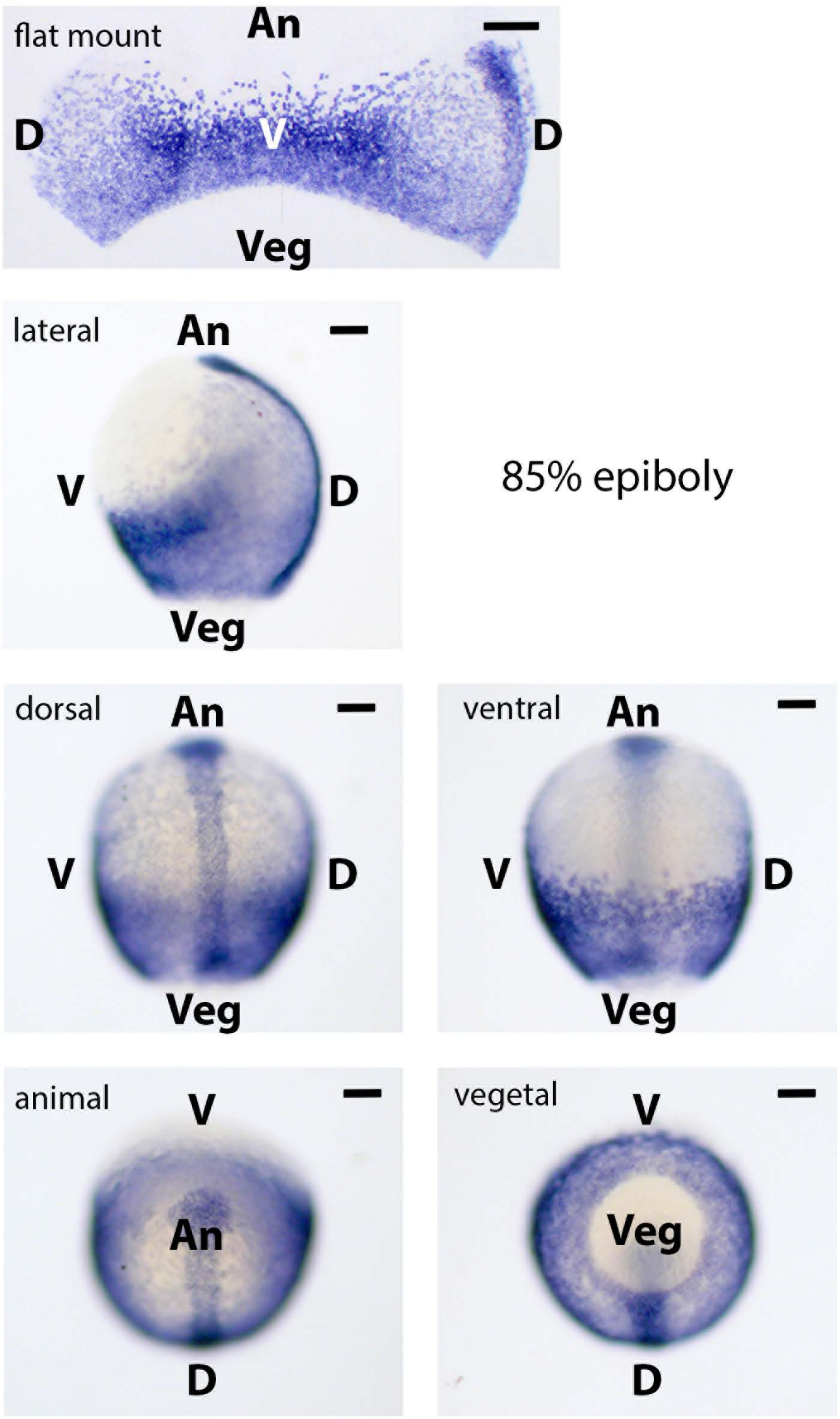

Supplementary Figure 1: Expression profile of *zulu*.
Detection of *zulu* transcripts by whole mount *in situ* hybridization is shown at the 85% epiboly stage in flat-mounted and unflattened embryos.
Abbreviations: An, Animal; Veg, Vegetal; D, Dorsal; V, Ventral.
Scale bar represents 200 μm in top panel and 100 μm in bottom wholemount panels

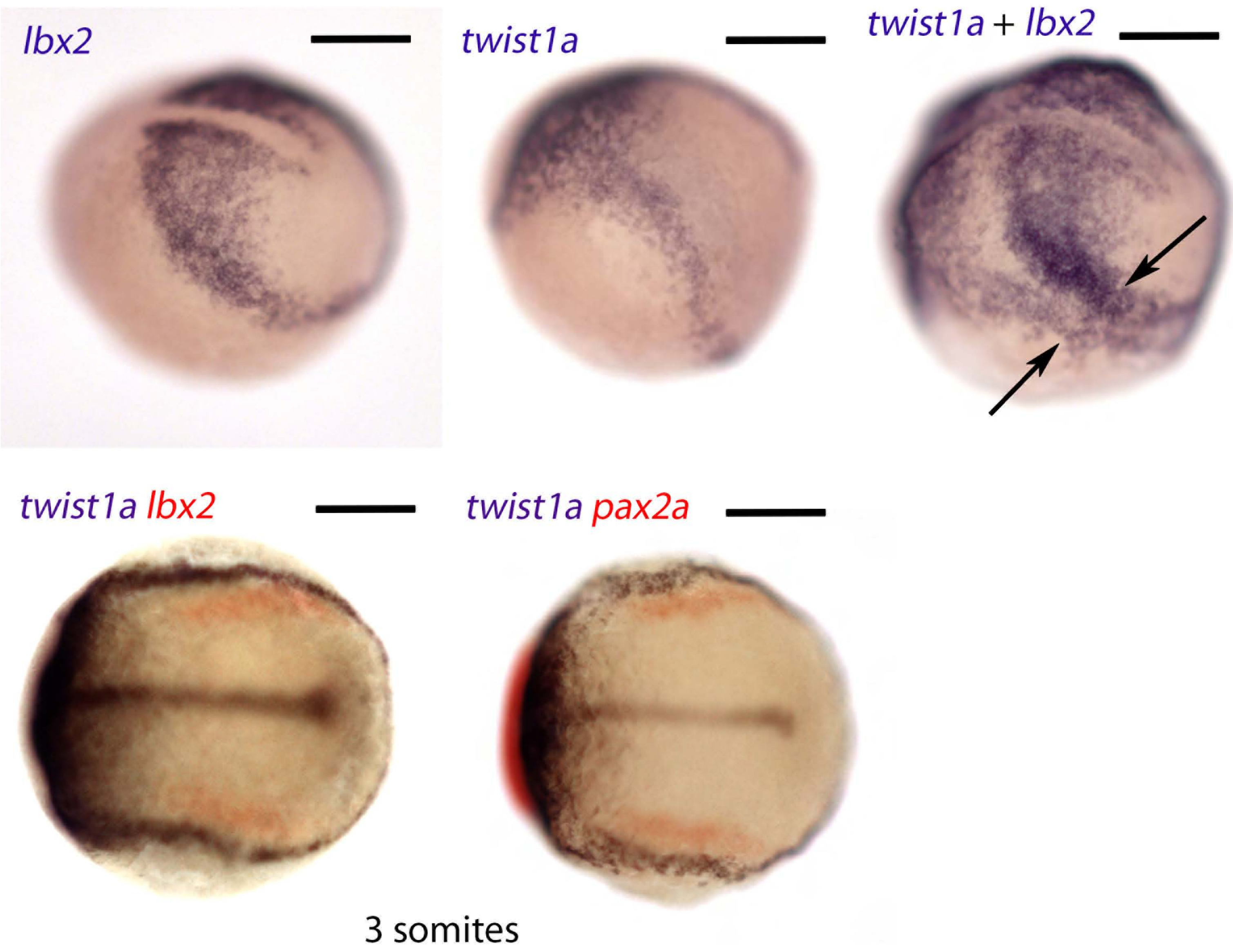

Supplementary Figure 2: Expression patterns of *twist1a, lbx2,* and *pax2a*. Upper panels show the expression patterns of *twist1a* and *lbx2* at late gastrulation/early somitogenesis by single (left and middle panels) and double (right hand panel) whole mount *in situ* hybridisation. Arrows indicate the non-overlapping *twist1a*$^{+}$ and *lbx2*$^{+}$ domains corresponding to presumptive lateral plate mesoderm and intermediate mesoderm, respectively. Lower panels show two color double stainings at the 3 somite stage for *twist1a*, *lbx2* and *pax2a*, as indicated. Scale bar represents 100 µm

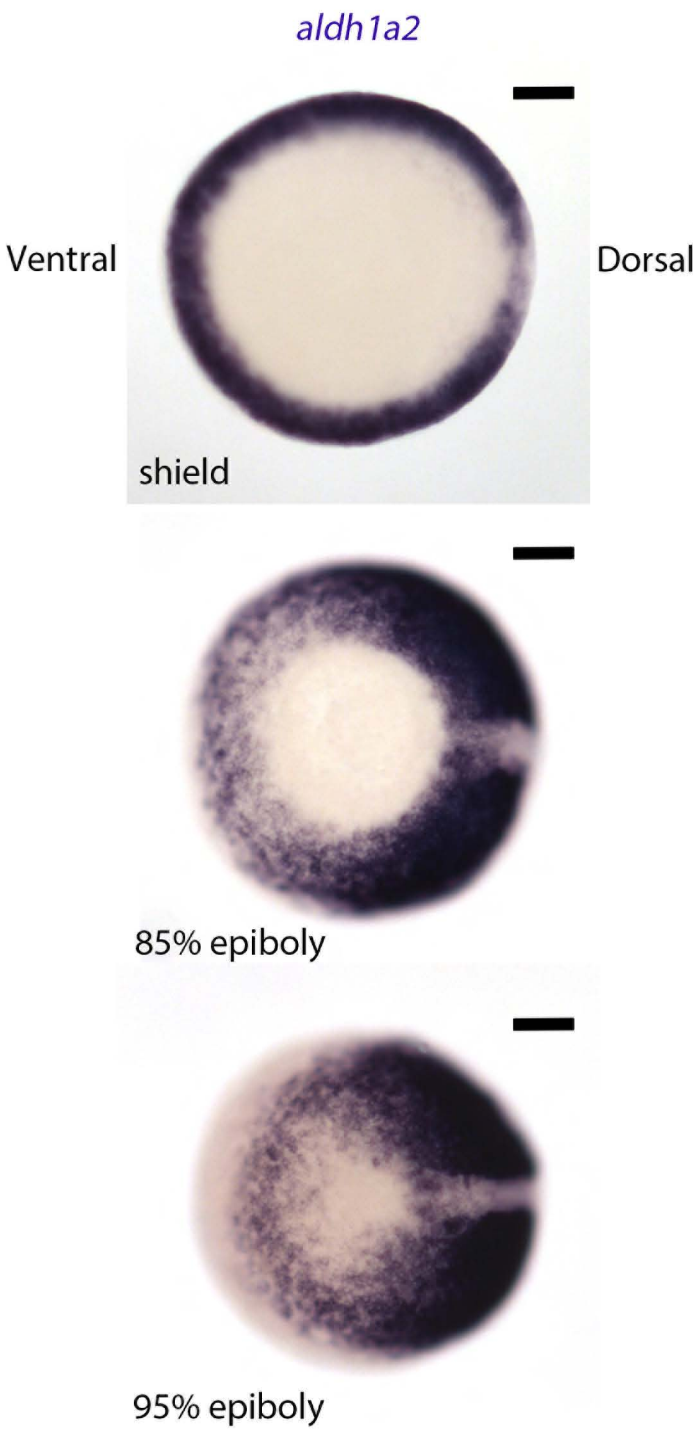

Supplementary Figure 3: Gastrula stage series for *aldh1a2* expression.
Detection of *aldh1a2* transcripts by whole mount *in situ* hybridization is shown at the shield, 85% epiboly and 95% epiboly stages. Embryos are viewed from the vegetal pole, with ventral to the left and dorsal to the right.
Scale bar represents 200 μm

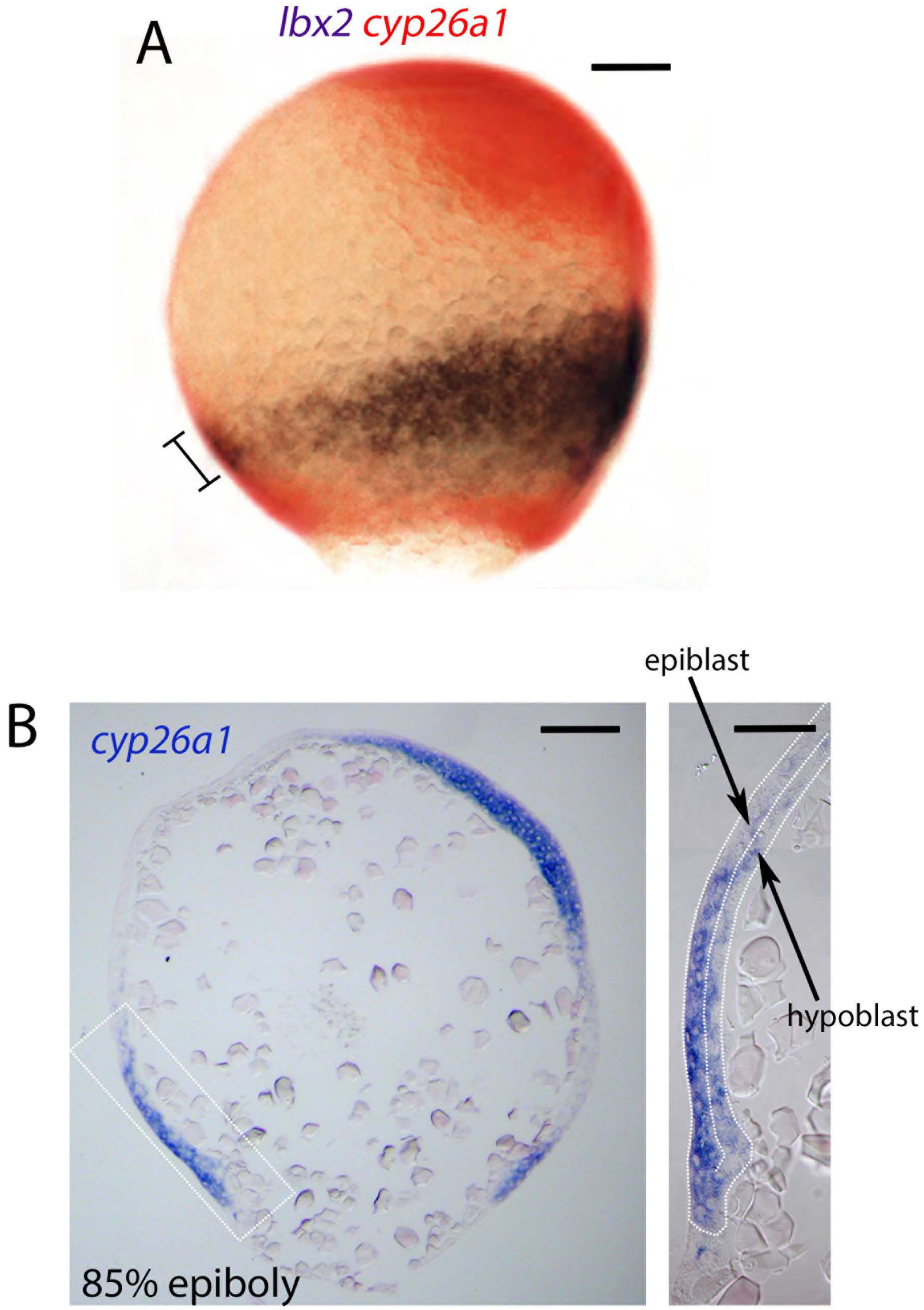

Supplementary Figure 4: Expression of *cyp26a1* and *lbx2*.
A) Detection of *lbx2* (purple/black) and *cyp26a1* (red) transcripts by whole mount *in situ* hybridization is shown at the 85% epiboly stage. The embryo is viewed laterally with the dorsal midline on the right. Bar indicates overlap between *cyp26a1* and *lbx2* in the presumptive lower PLM domain. B) Cross section of an 85% epiboly embryo stained for *cyp26a1* transcripts showing expression in the hypoblast and epiblast layers of the embryo. Scale bar in A) and low mag panel in B) represent 100 µm, scale bar in the inset high mag panel represents 50 µm

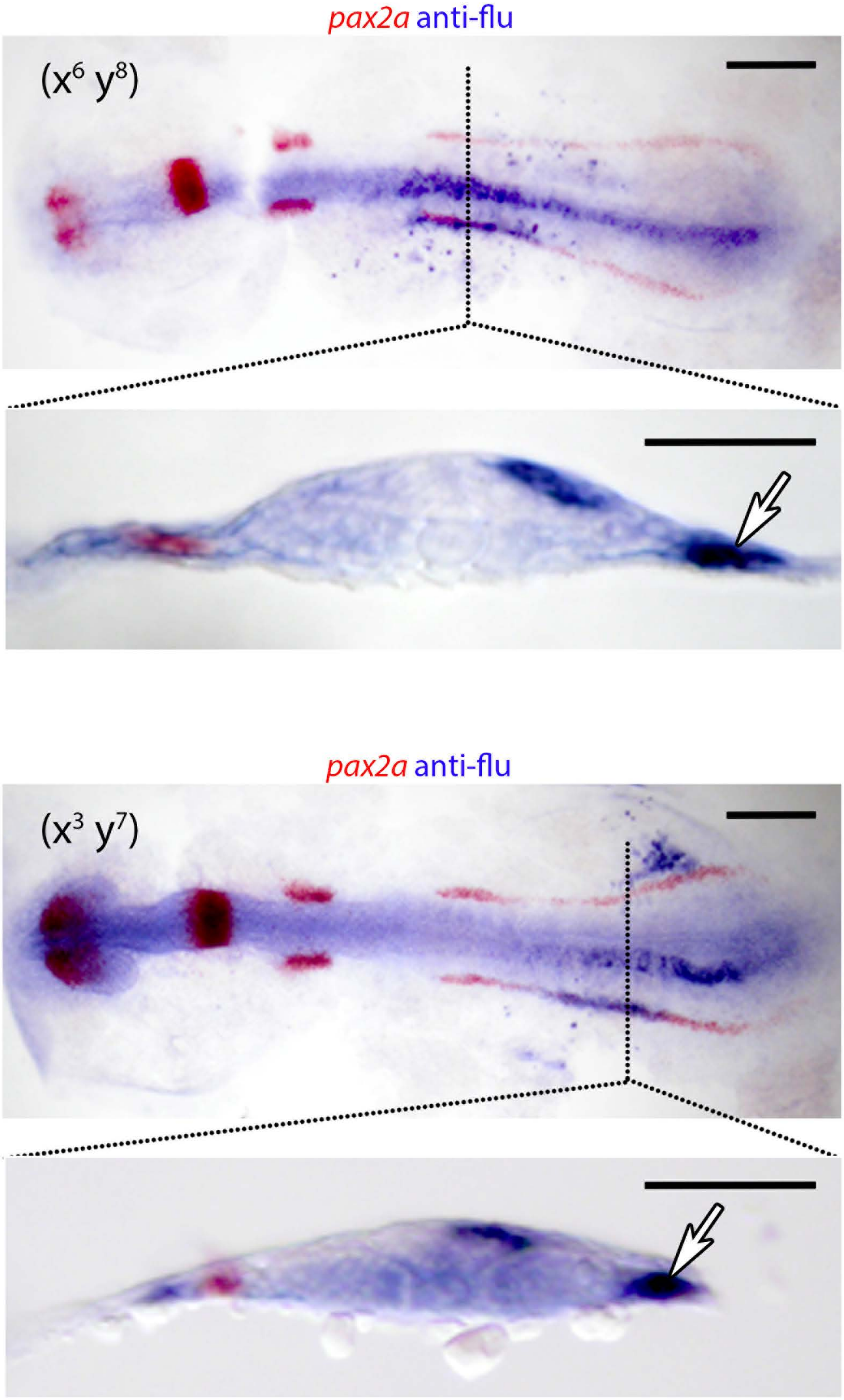

Supplementary Figure 5: Cross-sections of fate-mapped embryos double-stained for *pax2a* transcripts. Anterior and posterior nephron progenitors were lineage labeled in 85% epiboly embryos, examined by whole mount *in situ* hybridization at the 10 somite stage, and sectioned. Labeled cells contributing to the proximal and distal nephron were identified using an antibody to uncaged fluorescein (purple) and antisense probe for *pax2a* (red). Flat-mounted embryos are shown with anterior to the left, transverse sections are orientated with uncaged side to the right. Scale bars represent 200 μm

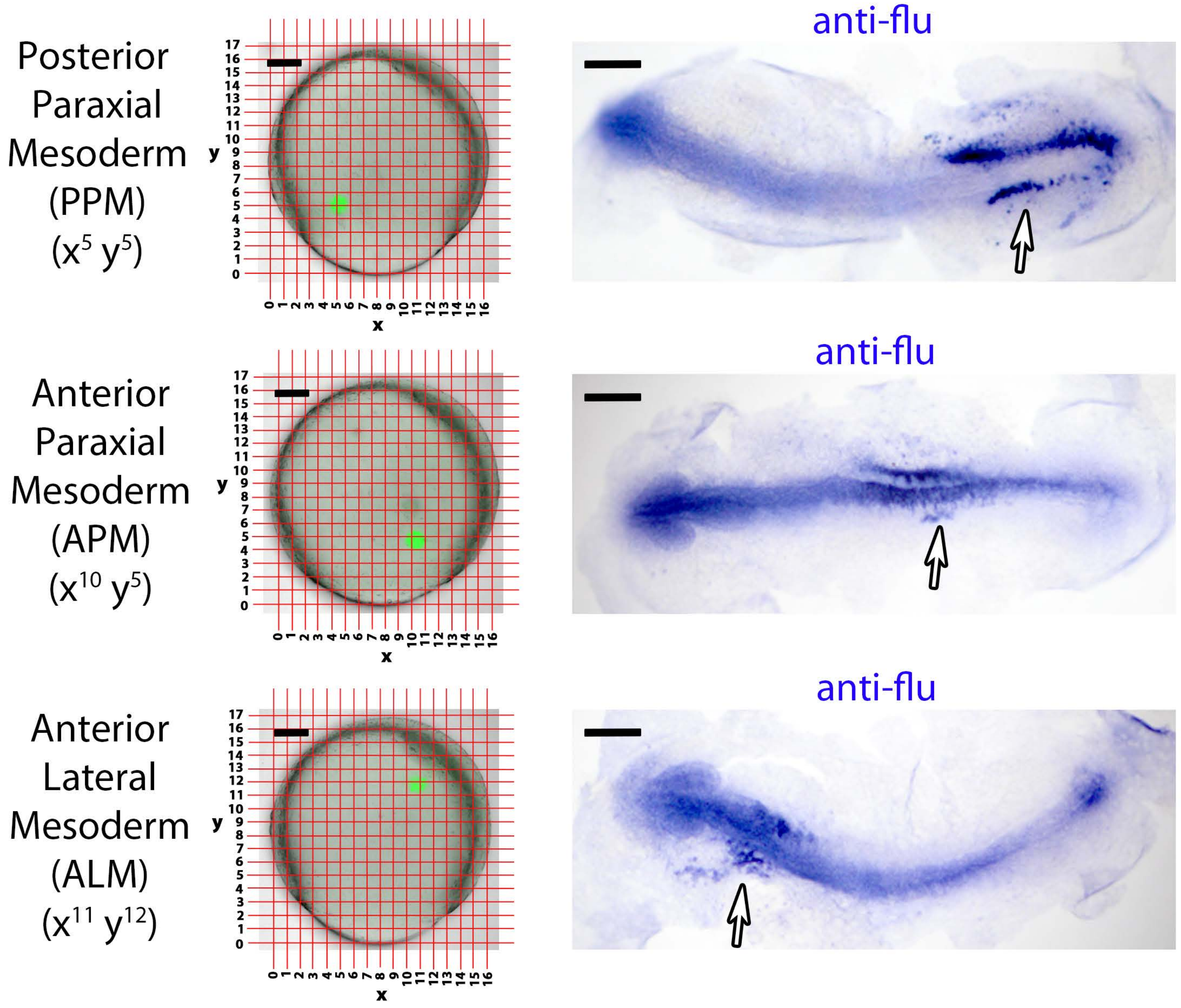

Supplementary Figure 6: Fate-mapping of the PPM, APM and ALM domains at 85% epiboly stage. Embryos at the 85% epiboly stage were uncaged at various co-ordinates on the Cartesian grid as indicated (embryos are orientated with animal pole towards the top and dorsal side to the right). At the 10 somite stage, uncaged cells were detected by whole mount *in situ* hybridisation. Embryos are flat mounted with anterior to the left. Scale bars in left panels represent 100 µm and in right panels represent 200 µm

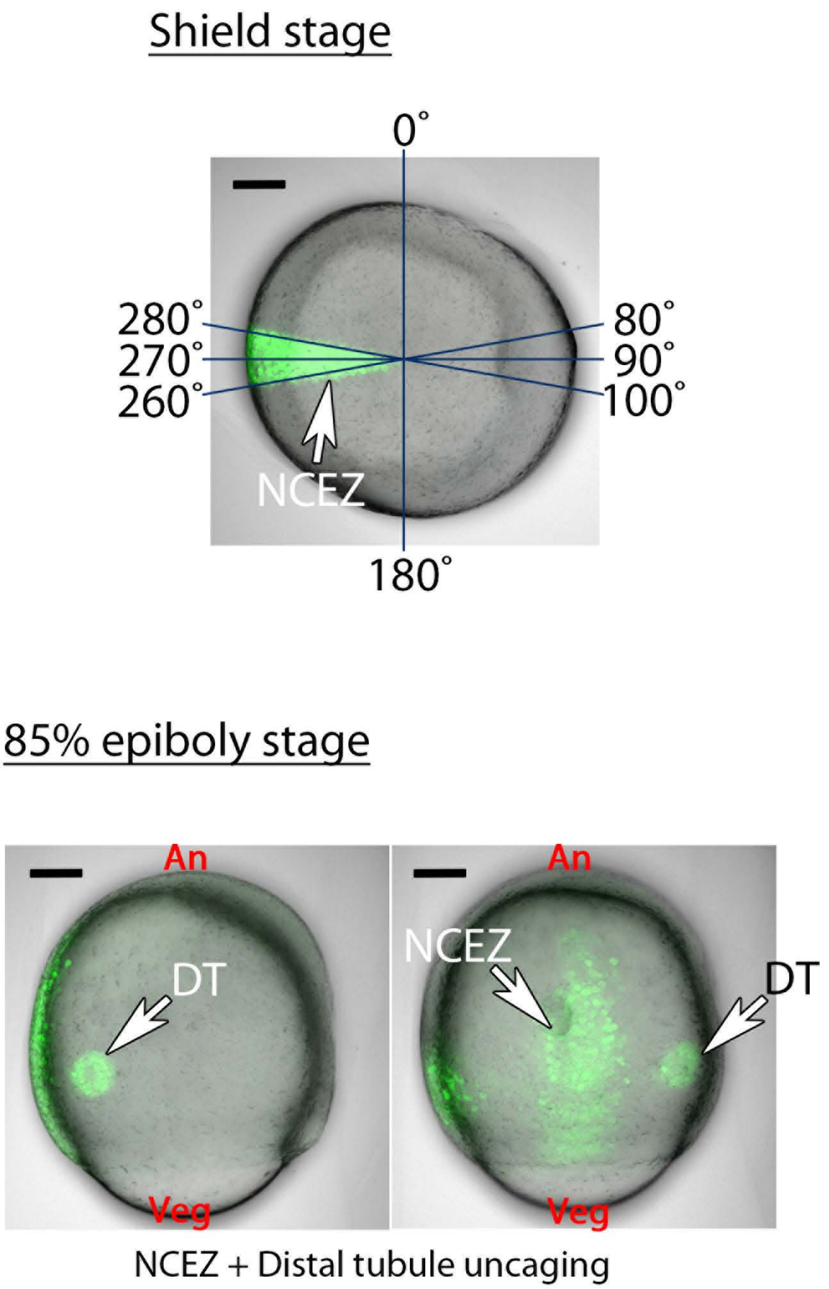

Supplementary Figure 7: Distal tubule cells are positioned outside the non-convergence extension zone (NCEZ). To test if distal tubule (DT) progenitors are positioned within the NCEZ, we uncaged embryos injected with a caged fluorescein dextran lineage label at the shield stage (6 hpf) in a position that encompassed the ventral-most 20° (immediately opposing the organizer). These embryos were left to develop to the 85% epiboly stage and then DT progenitors were labeled by uncaging at position (x^3, y^7). We find these progenitors are not positioned within the NCEZ. Top panels are dorsal views, bottom left panel is a lateral and bottom right panel is a ventral midline view. Scale bars represent 100 µm

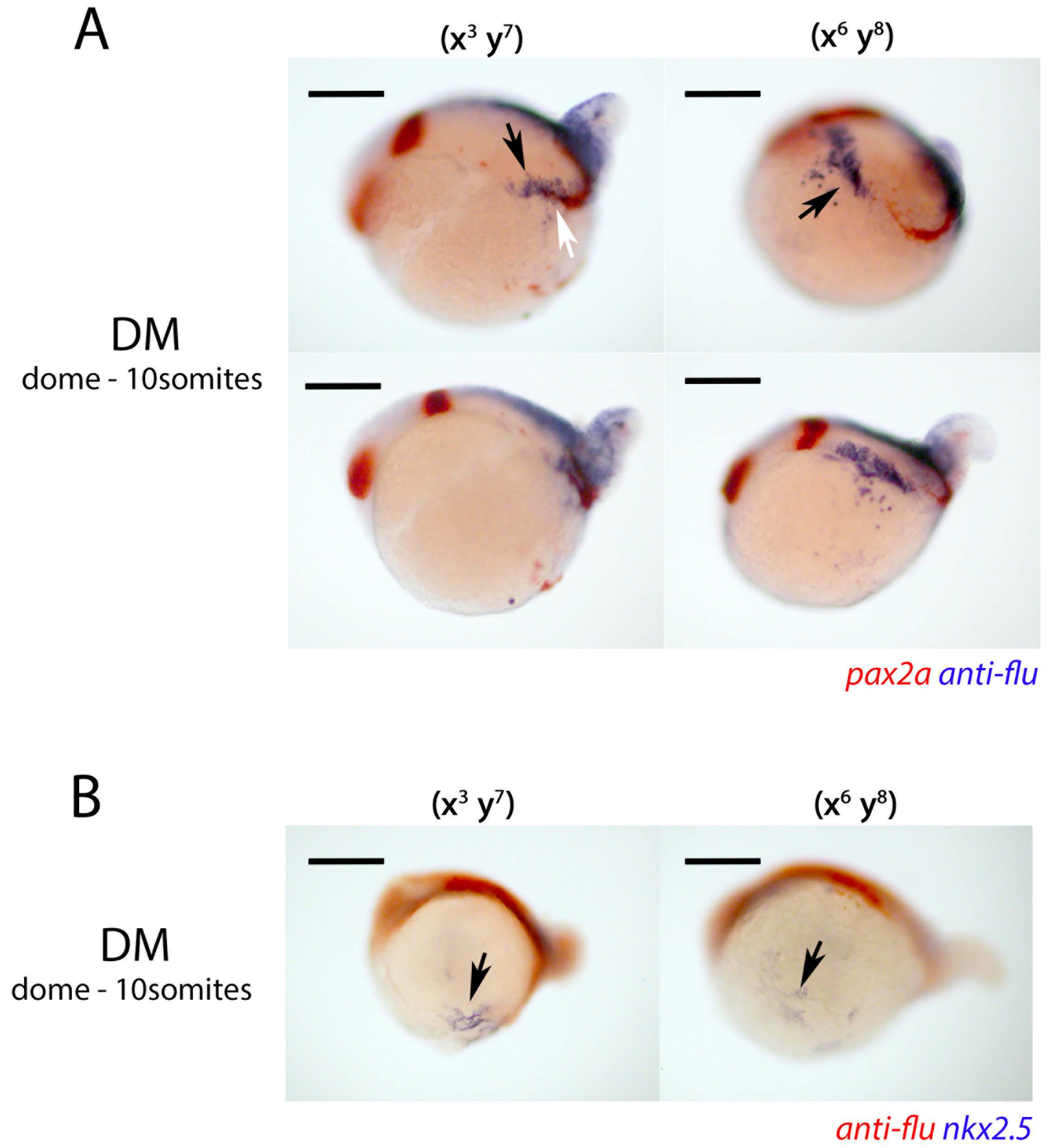

Supplementary Figure 8: Oblique and lateral views of DM treated embryos lineage labeled at 85% epiboly at positions (x^3, y^7) and (x^6, y^8). A) The same DM treated embryos shown in Figure 8B are shown here, with the top panels being oblique views and the bottom panels being lateral views. In the (x^3, y^7) labeled embryos the lineage tracer can be detected within the anterior-most region of the pronephric *pax2a*$^+$ domain (white arrow) as well as in a medial domain we presume to be paraxial mesoderm (black arrow). In the (x^6, y^8) labeled embryos, oblique views highlight the more anterior region of tissue (black arrow), which we predict is anterior paraxial mesoderm, that has been traced in these DM treated embryos. B) The same DM treated embryos shown in Figure 8C are shown here, but in lateral views where *nkx2.5*$^+$ heart progenitors can be better observed (black arrows). Scale bar represents 100 μm

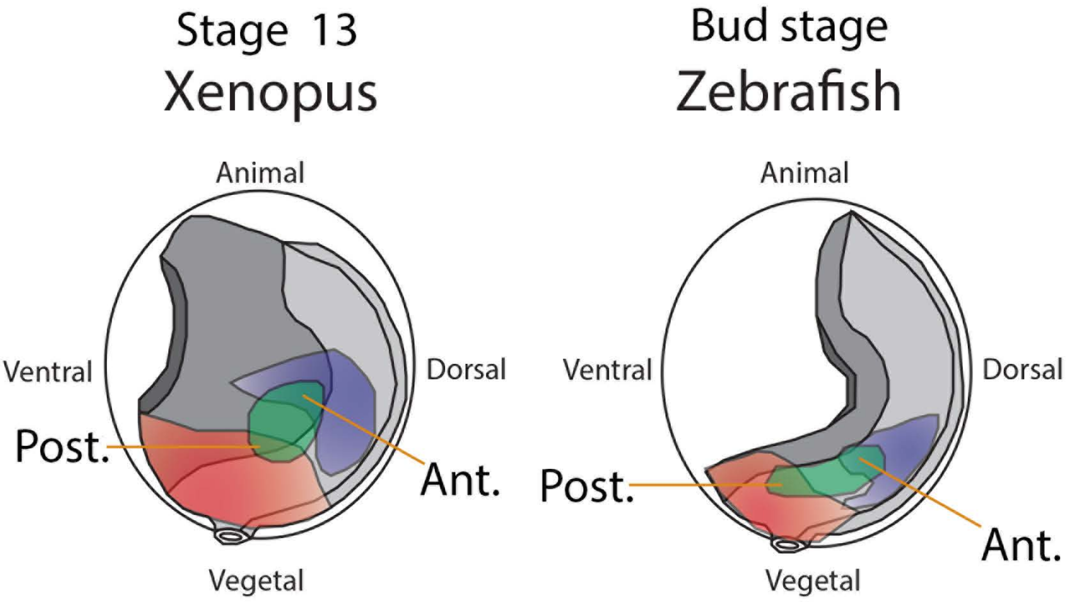

Supplementary Figure 9: Schematics comparing *Xenopus laevis* and zebrafish pronephric progenitor position in relation to *aldh1a2* and *cyp26a1* expression domains in the mesoderm. The mesoderm of both *Xenopus* and zebrafish contains *aldh1a2*$^{+}$ (blue) regions in the presumptive anterior trunk region at stage 13 in *Xenopus* and the corresponding bud stage in zebrafish. Both model organisms also posess a *cyp26a1*$^{+}$ posterior domain in the ventral mesoderm (red). The approximate positions of the pronephric progenitors are shown (green). In both organisms the pronephric intermediate mesoderm bridges the RA source (*aldh1a2*$^{+}$ domain) and the RA sink (*cyp26a1*$^{+}$ domain). Abbreviations: Ant., Anterior; Post., Posterior. The *Xenopus* schematic was modified from Supplementary reference 1 with the permission of Cold Spring Harbor Press.

Supplementary References

1 D., S. C. Gastrulation: From Cells to Embryo. Cold Spring Harbour Press (2004).
